# Self-directed learning in Orthopaedic trainees and contextualisation of knowledge gaps, an exploratory study

**DOI:** 10.1186/s12909-024-06269-2

**Published:** 2024-11-16

**Authors:** Ahmed Maksoud, Farah AlHadeed

**Affiliations:** 1 University of Dundee, Dundee, Scotland; 2 Peninsula School of Medicine and Dentistry , Exeter, Peninsula UK

**Keywords:** Medical, Self-directed learning, Surgical, Resident, Orthopaedics, Autonomy, Self-regulated learning, Postgraduate

## Abstract

**Background:**

Self-Directed Learning (SDL) is a subjective concept with no universally agreed definition. The aim of this project was to obtain the perspectives of Orthopaedic trainee registrars on SDL.

**Methods:**

Seven Orthopaedic trainees in the Southwest Peninsula region were recruited in person by the primary author as an Orthopaedic trainee on the same programme as the participants. A one-hour Microsoft teams video interview was arranged at a mutually convenient time exploring several topics including learning resources, experiential learning, learning strategies, training programme requirements and assessment. The interviews were audio recorded and transcribed verbatim. Transcripts were coded using Corbin’s and Strauss’s published coding method and analysed to develop a model of SDL in this training context.

**Results:**

Six learning stages were identified from the coded transcripts and these stages were like Knowles’s stages of SDL. Participants felt less autonomous during the identification of knowledge gaps, goal setting and evaluation of learning stages. Participants perceived to have more control over the selection of strategies, and learning stages of SDL. The factors which influenced autonomy at various stages of SDL included supervisors, experiential learning opportunities, service provision competing with learning opportunities, training programme requirements and the power dynamic between trainees and supervisors. Self-advocacy by the trainees for experiential learning opportunities and for their progression was an additional action relevant to SDL that was evident. Online learning resources such as Orthobullets.com, YouTube videos, external revision courses, collaboration and webinars encouraged more autonomy with SDL.

**Conclusions:**

Although the United kingdom’s Trauma and Orthopaedic curriculum is described as trainee led and SDL is encouraged, in practice Orthopaedic trainees felt limited autonomy with the various stages of SDL due to external factors including their level of experience and the training environment. Trainees’ experiences around self-advocacy highlighted the importance of a collaborative and supportive learning culture emphasising dialogue, receiving high quality feedback, and openness for successful progression.

## Background

I, the primary author, completed this project on Self Directed Learning (SDL) as part of a Masters in Medical education during my Year 6 Orthopaedic training year in the Peninsula deanery. Although didactic teaching is provided by the Peninsula training programme, it is not exhaustive. Extra reading and revision are needed to satisfy the knowledge requirements for the Fellowship of Royal College of Surgeons (FRCS) exam. Therefore, SDL is needed for comprehensive coverage of the curriculum. However, in an Orthopaedic training context, SDL is likely to be influenced by many external factors including educational culture of the institution, expert guidance, experiential learning opportunities and trainee factors such as past experiences, study skills, motivation, and personal attributes. Thus, SDL should not automatically signify complete learner autonomy. How trainees gain knowledge and skill via courses, expert guidance, reflection, and experiential learning opportunities is an important part of SDL which I felt was worth exploring. This question prompted the research project which aimed to interview trainees on the same programme I was undertaking to summarise their experiences around SDL. SDL can be approached in several ways; as an attribute of the learner, or a process the learner undertakes or an instruction method. Herein, SDL was approached as a process the learner follows and it is assumed that learners are autonomously seeking the best strategies to maximise their learning. Using trainee perspectives to explore both personal and external institutional factors affecting SDL can offer important insights which can be useful to consider when designing and delivering postgraduate medical education curricula.

### Defining SDL

The Trauma and Orthopaedic Surgery curriculum, as set out by The Intercollegiate Surgical Curriculum Programme (ISCP) in the UK, stresses trainee-led learning, with a focus on Self-Directed Learning (SDL) [4]. Trainees must grasp the fundamental principles of Orthopaedic surgery applicable across all subspecialties to reach competence and safety as consultant Orthopaedic surgeons [4]. The Peninsula Trauma and Orthopaedic Training Programme website emphasises the importance of extra reading and revision beyond its fortnightly teaching sessions to fulfil the knowledge requirements for success in the Fellowship of Royal College of Surgeons (FRCS) exam [31]. Thus, SDL is essential for comprehensive learning.

Knowles, a prominent scholar of adult education, first used the term SDL. He described it as a process in which adult learners find their learning needs, find resources and strategies to achieve their goals, and evaluate their progress, either independently or with expert guidance [22]. The Orthopaedic curriculum views SDL as a proactive learning approach where trainees set up study groups, engage in journal clubs, utilise peer and formal learning, seek feedback, and engage in reflection to guide their learning endeavours [4]. Central to SDL is the concept of authentic control, wherein learners hold authority over educational decisions [7]. The determination of goals and resources, selection of effective methods, and assessment criteria are the responsibility of the learner. While the curriculum aids in finding learning needs through learning goals, it however, offers minimal guidance on the best strategies to attain them. Herein, we explore how Orthopaedic trainee surgeons utilise learning resources and what strategies they use to address their learning goals.

### Researching SDL

Knowles highlighted a set of assumptions about how adults learn. These assumptions include the belief that adults have a self-concept of independence, are motivated to learn only material relevant to their needs, adopt a problem-centred rather than subject-centred approach to learning, are influenced by their prior experiences, show internal motivation, and require understanding of the reasons behind learning something [25]. The term SDL has led to confusion in the literature and has been used in several ways: to describe an attribute of adult learners, a learning process, or an instructional approach following andragogical principles. Regardless of the interpretation, there is a common emphasis on granting learners autonomy and responsibility over their learning process in contrast to traditional pedagogy where learners rely entirely on teachers. SDL is often used interchangeably with terms such as self-planned learning, self-education, self-teaching, autonomous learning, autodidaxy, independent study, and open learning [32].

Subjectively around the extent of autonomy or responsibility a self-directed learner should or can realistically achieve, makes SDL an elusive concept with disagreements on its definition and utility [33]. It's important to note that SDL does not imply lack of reliance on an educator or teacher. Educators often play a crucial role in introducing learners to fundamentals and act more as facilitators or sources of skill [12, 23]. Knowles's model of SDL considers the instructional context and does not diminish the educator's role [26]. Brookfield highlights learner autonomy while acknowledging that learners may still seek guidance from experts in a "field-dependent" form of SDL [5, 6]. In this form of SDL, learners consciously opt to follow expert advice or utilise instructional courses due to factors such as convenience, learning style, or pace [24].

Some scholars advocate for a stricter definition of SDL, asserting that true self-directedness involves learners self-assessing, a skill often underdeveloped in healthcare professionals [34]. Furthermore, evidence for the effectiveness of SDL is limited, with only a few randomised controlled studies available [32]. Additionally, scales for assessing self-directedness lack standardization, with the most renowned scale, the Self-Directed Learning Readiness Scale (SDLRS), being derived from a Delphi study of consensus rather than empirical evidence [18].

### Challenges when researching SDL

We faced significant challenges whilst researching Knowles’s linear and cyclical model of SDL within the clinical environment of Orthopaedic training. Briefly, these are the need for expert guidance and the importance of experiential learning opportunities. The need for expert guidance and experiential learning arises due to the shift in medical education assessment towards outcome-based approaches, where emphasis is not solely on factual knowledge but increasingly on complex decision-making and depth of understanding to simulate real-life complexities and ensure best patient outcomes [17]. Dewey’s pragmatic teaching theory asserts that effective instruction should be grounded in experience to promote authentic learning [13]. Indeed, junior trainees have shown a preference for guided case discussions over unguided ones [1], and SDL has been found to be less effective in junior trainees [32]. In certain clinical contexts, SDL must occur opportunistically and may not follow a predictable cyclical process that is entirely controlled by the learner [3]. Experiential learning in the workplace is unpredictable and may occur in sporadic leaps and bounds rather than in the linear and incremental fashion suggested by Knowles’s model of SDL. Therefore, when investigating SDL in Orthopaedic trainees, a significant departure from the principle of authentic control [7] is needed.

Furthermore, a key consideration lies in the considerable influence of the institution and the surrounding educational culture on SDL, which influences its success. For instance, extensive research in medical education by Berkhout and Dornan highlighted how student and resident workplace learning is significantly influenced by the workplace environment [14]. Greveson and Spencer [16] discussed how motivation and receptiveness to SDL vary depending on context, including social, cultural, educational settings, past experiences, relevant study skills, and self-concept. A recent conference proceeding outlined consensus findings from the faculty of eight American medical schools to define key elements of SDL. These elements include personal attributes such as resilience and curiosity, institutional environment factors such as tolerance of uncertainty and psychological safety, as well as assessment and faculty development aspects like questioning techniques [16]. In summary, the multitude of subjective approaches to SDL makes researching it a challenging process, especially when transitioning its application from classroom-based education to the unpredictable and complex clinical environment. For this reason, we employed an iterative Grounded Theory approach to the literature review, focusing on elements of SDL that Orthopaedic trainees find relevant to their clinical training.

### Introducing the researchers

Considered an insider researcher, as primary author I belonged to the same group as the research participants, as we all worked in the same institution and attended the same Orthopaedic Training Programme [27]. Like my colleagues, I completed core surgical training and medical school in the UK, affording me convenient access to participants whom I regularly meet at work. I was well-acquainted with the internal learning environment in Orthopaedics and the various challenges to learning, often engaging in spontaneous and informal discussions with colleagues about learning during regional training events. The second author has offered useful and independent insights to aid the primary author in interpreting the results and the write up of this publication. The tables and the raw quotes in the results section were reproduced from a Master dissertation completed by the primary author over the period April 2021 to May 2023.

## Methods

### Ethics approval

The study proposal was submitted to and approved by the Schools of Medicine and Life Sciences Research Ethics Committee (SREC) at the University of Dundee (REC Number 21/60).

### Participant recruitment

Eleven participants were recruited in person by the primary author during regional training days from January to July 2022. They were emailed an information sheet, consent form, and an invitation to a Microsoft Teams interview. Seven participants responded. Informed consent for participation in the study was obtained from all seven participants and they were subsequently interviewed. The interviews were audio-recorded with consent and transcribed verbatim using Otter.ai. Five interviewees were post-FRCS trainees and two were pre-FRCS trainees of varying seniority (Three ST8s, Two ST7s, One ST4 and One Post-training, two participants were females the rest male). Data analysis and literature review were conducted alongside the interviews, refining the questioning technique with each session.

###  Coding and data analysis

**Fig. 1:**
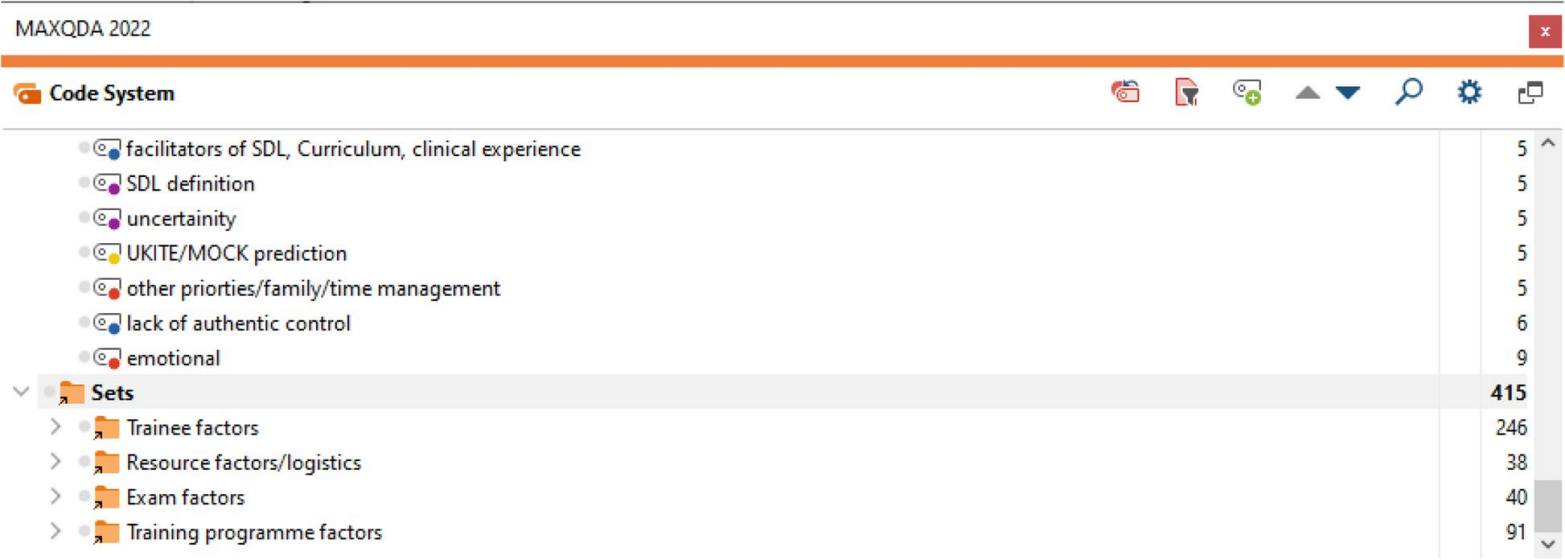
MaxQDA interface, 415 line-by-line codes

**Fig. 2:**
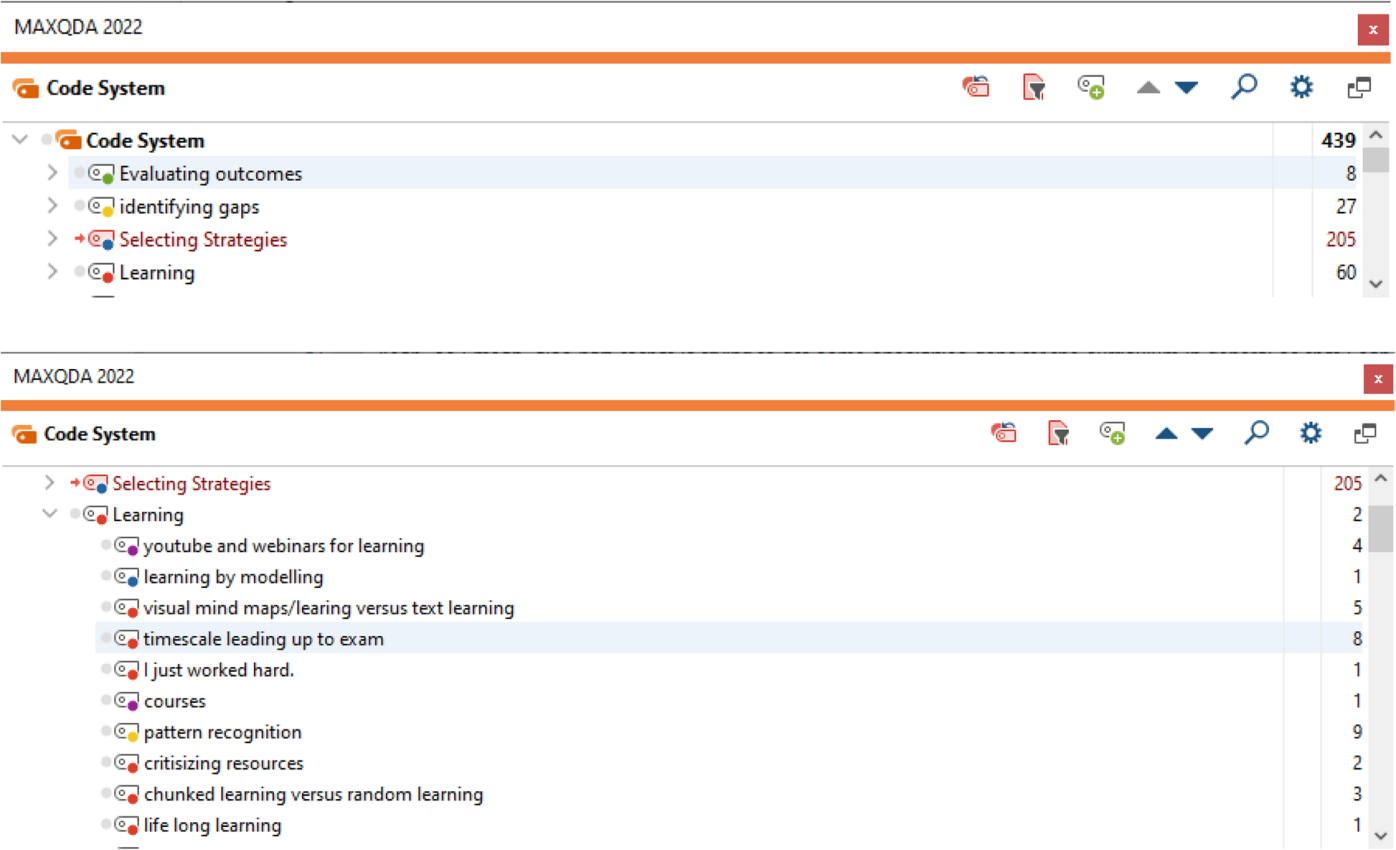
MaxQDA, Actions as main codes, conditions and properties as subcodes

In the first data analysis stage, line-by-line coding was used to classify the data into sets (see Figs. 1 and 2). This approach helped interaction with the data and ensured the research met grounded theory criteria: fit and relevance. However, it also fragmented the data into seemingly unrelated categories, complicating model building for SDL [8, 9].

In the second stage, Corbin and Strauss's coding principles, focusing on actions and interactions, were applied. All transcripts were re-coded to denote learner actions, using MaxQDA to aid the process. Action codes were sub-coded with properties or factors affecting those actions, many of which were found in the first line-by-line codes. This approach gradually linked all codes into a cohesive model [10].

The coded learner actions were tabulated according to specific actions showed by the quotes. The raw data was colour-coded to describe influencing conditions, which were then rearranged by colour code to aid further analysis. MaxQDA significantly eased this process by simplifying the analysis and interaction with the extensive transcripts.

### Interviews

A prompt sheet of topics to explore with the participants was developed from an initial literature review and structured according to the multiple factors affecting training under general headings (Trainee factors, Training Programme factors, Resource factors and Assessment factors). A similar strategy to that used by Wasserman et al. [38] in their study on homelessness was adopted where the literature review was guided and focused on areas of relevance by the data collected. The prompt sheet in turn was continuously refined and developed with each interview as specific topics of interest were raised, explored in the literature and with each subsequent participant. The interview prompt sheet of topics is included as a supplementary document.

## Results

Table 1 summaries each of the six learner actions, their properties and influencing factors. Detailed findings about each action are illustrated with quotes. Participants are denoted P1 to P7 according to the chronological order of their interview which took place over the period January to July 2022.

**Table 1:** Summary of all identified actions, their properties and influencing factors

Action	Property	Influencing factor			
		**Learner factors**	**Assessment Factors**	**Resource Factors**	**Programme factors**
Identifying knowledge gaps	Opportunistic versus Structured	Past skills Professional responsibility	Time left to the exam	Instructional courses	Rotation design
Goal setting	SMART	Priorities Locus of control Motivation	Time left to the exam	Commercial resources such as Orthobullets.com	Consultant support
Selecting Strategies	Versatility versus Commitment	Trust Pragmatism	Time left to the exam Exam format	Structure of the resource Engaging resource	Advice from peers and from supervisors
Learning	Depth, Type, Intensity, Active versus Passive	Learner’s judgement Motivation Reflexivity Study skills	Time left to the exam Exam format	Wordiness of resource Technology	Power balance Service provision/time available for teaching and learning
Evaluating outcomes	Degree of conformity with received feedback	Self-concept Pragmatism Growth versus Fixed mindset	Perceived validity and reliability of the assessment	N/A	Feedback style Feedback promoting a growth mindset versus fixed mindset
Negotiating	Assertiveness or Vigor	Expectations Assertiveness Self-concept Beliefs	N/A	N/A	Resources Expectations Trainer resources Trainer ability

### Identifying gaps in knowledge

For participants, the main challenge to successful SDL was that they *“do not know what [they] need to learn”*. External influences were the predominant method by which they became aware of their knowledge gaps. For example, prompts from consultant supervisors, a clinical encounter, regional training, or the annual mock exam *“stimulate the SDL”.*

Compared to the opportunistic nature by which knowledge gaps were identified during the earlier years, in the lead up to the FRCS exam more methodical methods were used. Participants *"changed Tactics"* and they *“stopped focusing on the clinical placement,”* to clarify *“the whole scope of the curriculum”* and made themselves *“aware of what potentially [they] could be examined on.”* Participants used instructional courses and Orthobullets which helped them identify gaps in knowledge while before they did *“not know that [they had] to know”* about certain topics.

Trainees appreciated that they could not *“cover everything to a high level of depth”* and some *“never truly believed”* that they will “*know enough.”* Participants needed to gauge *“how much knowledge is enough”* and needed to *“set limits”* to prevent themselves against going down a *“rabbit hole.”*

Participants felt overwhelmed with the amount of knowledge to cover. When running out of time, they either *“started picking”* high yield topics or used *“random MCQ practice”* to find *“any drastic knowledge gaps”*.

Table 2 summarises the factors which influenced the process of identification of knowledge gaps.

**Table 2:** Factors which influence the identification of knowledge gaps

Property	Influencing factor				Example Quotes
	**Learner factors**	**Assessment Factors**	**Resources Factors**	**Programme factors**	
Structure (Methodical versus Opportunistic	Past skills Pragmatism Personal life Professional responsibility	Exam Time leading up to the Exam	Instructional courses Commercial programmes (Orthobullets)	Consultant input Organisation of the rotations/teaching programme	*P5: “I went through Rishi Dir’s videos (online instructional course), I knew that they were a thing that I had to learn whereas before that point, you don’t know that you have to know about the history of how theatres are designed”* *P3: “it’s sort of more experiential, directed by things that trigger you to learn a topic or based on your experience, which is like your clinical experience or the boss telling you about something.”* *P5: “Now I'm not going to do them all, but I'm going to read most of the topics in some kind of structured way so that in my head, I'm basically creating a filing system and I kind of know where you know my foot and ankle information is, my like basic science information is, my hips and knees and stuff is.”* *P3: “But that quickly became quite overwhelmingly massive in terms of the programme that I've chosen, so in the end, it became a bit more random.”* *P1: “I started picking. When I realised, I couldn’t keep up”*

### Goal setting

The trainees had to prioritise between exam preparation and advancing their surgical portfolio objectives. Selecting a goal and sticking to it was not easy due to *“limited control”* that participants had over their time and training requirements which *“can come in and interfere with”* goal setting. Whether or not a goal was pursued was dependent on its outcome being *“motivating enough”* and any competing goals to *“tick off as part of the syllabus along the way”* such as completing a *“100 hip replacements”.* Self-image maintenance motivated P5 to study for the yearly mock or UKITE as they did not want to *“look below [their] level of training”* or *“look stupid”*.

P5: *“Again, that wasn't my priority at the time, you know when it comes down to the mock exam. But the yearly mock exams definitely weren't motivating enough as a way of me trying to learn more orthopaedic knowledge. There was too much other things to tick off as part of the syllabus along the way”.*

Goal setting was influenced by supervisors as participants were “*signed off”* to sit the exam. Time was provided by the supervisors for participants to revise as they *“appreciated how horrible”* revision time was for the exam. Supervisors relaxed expectations of audit, research and allowed them time to focus on the exam. Annual leave and study leave were used to revise for the exam.

Orthobullets provided a *“structured* overview” or a Master plan of the syllabus that made it easier to set goals. Several participant accounts echoed this.

Participant one:* “So it didn't feel like awful, I was enjoying it and I had a goal that I was heading towards. And I liked having a goal” (Feelings/feedback on Orthobullets.com).*

Table 3 summarises the factors which affected goal setting.

**Table 3:** Summary of factors which affect goal setting

Property	Influencing factor				Example Quotes
	**Learner factors**	**Assessment Factors**	**Resources Factors**	**Programme factors**	
Specific Measurable Achievable Realistic Timely (SMART)	Priorities Perceived control Self-image Motivation	Time leading up to the Exam Exam Format	Technology offering a “Master Plan” such as Orthobullets	Consultant support Unpredictability/lack of control Entrustment Annual leave Study Leave Training priorities	*P5: “I took five days study leave before the first one and before the second one and an additional week of annual leave before the second one”* *P6: “Because there’s limited control, you can plan to do some things, but there’s a lot of other things than can come in and interfere with that”* *P1: “I think SMART goals…they don’t account for things you don’t have control over, there’s so many things in clinical practice, in professional life* P5: “When you apply for it [the exam], you’ve got to get references, which basically is other people saying I back *** to pass the exam. I think he’s good for it and you’re like, well, it’s not only myself that I’ve got to prove to. I’ve got these other people that have put their word on the line that this guy is going to smash it and I’ve got to do it and people are watching me” *P5: “my motivation was like; I don't want to look below my level of training. You know, I didn't want to like look stupid”*

### Selecting strategies

Participants were uncertain about the best study strategies. Participants had to *“trust the advice*” of predecessors and to *“sift through all advice”*, choose from *“a vast load of resources”* and settle for one strategy or one resource. Participants preferred learning in chunks because random review of concepts made it *“difficult to link concepts together”* while chunked learning reinforced knowledge and performance improved.

### Influence of time pressure

Study strategies were also influenced by the amount of time leading to the exam. During periods without exams, trainees often aimed to thoroughly understand a specific subject rather than practicing random questions across a broad curriculum, P3: *“so in the end, it became a bit more random.”* In the final weeks before the exam, time constraints led some participants to address blocks of questions across the entire curriculum, while others focused on “*picking*” high-yield topics, accepting that they would not be able to cover everything.

As the exam date approached the need to identify knowledge gaps became a priority, leading trainees to adopt more efficient methods, such as practicing targeted questions and utilising curriculum overviews like Orthobullets. P1: *“I started picking. When I realised, I couldn’t keep up”.* Trainees have also spent more time reviewing questions and less time reading. *P5: “And it kind of got to the end and I was like right. I need to rationalise things now because I'm not going to cover everything. So, I basically then started spending less time reading & more time just doing the questions”.*

Due to exam pressure, there was a shift away from “learning on the job” with more emphasis on a targeted overview of the overall curriculum. For example, P1 described a clear change in their learning strategy as the exam approached. Initially their “*philosophy through all of my training has been study what I'm doing on the job. So those are the things that I'm seeing in work and the things that I should be reading about. So, I've always taken the view that if I'm rotating through foot and ankle, I need to read about foot and ankle, I need to go to clinic, see what it is that I need to know more about and do my Further reading on those topics”*. However, *“I think in the lead up to the exam, that changes dramatically, as you suddenly have to identify all the gaps in your knowledge, so in the lead up to the exam, I stopped focusing on my clinical placement, and I started focusing on finding the gaps and filling those, so I changed tactics”.*

Table 4 summarises the influencing factors on the selection of strategies.

**Table 4:** Influences on learning strategies

Property	Influencing factor				Example Quotes
	**Learner factors**	**Assessment Factors**	**Resources Factors**	**Programme factors**	
Commitment/ Versatility	Trust Beliefs Critical reflection Realism Pragmatism Previous education	Time leading up to the exam Exam format	Structure of the resource How engaging the resource is Popularity Legitimacy	Peers Advice from supervisors Legitimacy	*P5: “I just need to make a decision. Stick with it. That is hard and there’s no answer about what the right thing is to do”* *P5: “I know a few people have used Orthobullets, and they've used the set like syllabus, like a 21 week, like question bank thing. So, I was like, right. Without looking at it in too much detail; someone else has done it. I trust them”* *P5: “And it kind of got to the end and I was like right. I need to rationalise things now because I'm not going to cover everything. So, I basically then started spending less time reading & more time just doing the questions”* *P3: “So certainly for part one of the exam, I did almost no reading, I just did lots and lots of practice questions, which obviously there’s some reading in that but doing practice questions I think actually directs you to what you probably need to know”* *P3: “I suppose that maybe you won't feel comfortable; not to bother reading much. But I know it is useless really “* *P5: “You’ve kind of just got to get committed and not listen to everyone’s else’s advice because that’s when it gets really stressful. I don’t want to spend like 1000s and 1000s of pounds on all of these courses”* *P1: “I need to reinforce [so that], it sticks in my head. And I've learned through med school that doing your assessments or your reading always went hand in hand with questions”* *P1: “The philosophy through all of my training has been study what I'm doing on the job. So those are the things that I'm seeing in work and the things that I should be reading about. So, I've always taken the view that if I'm rotating through foot and ankle, I need to read about foot and ankle, I need to go to clinic, see what it is that I need to know more about who my Further reading on those topics”* *P1:“I think in the lead up to the exam, that changes dramatically, as you suddenly have to identify all the gaps in your knowledge, so in the lead up to the exam, I stopped focusing on my clinical placement, and I started focusing on finding the gaps and filling those, so I changed tactics”*
Creativity	Versatility Flexibility Confidence Ability to manage stress Reflexivity Realism Pragmatism	Time leading up to exam Exam format	Popularity Legitimacy	Peers Advice from supervisors Legitimacy	*P3: “But that quickly became quite overwhelmingly massive in terms of the programme that I've chosen, so in the end, it became a bit more random.”* *P2: “Yeah, again. I don't know how he learned but I think he just did you know scattergun random questions”* *P1: “A lot of people have advised me, that the best practice for that is to practice MCQs”* *P6: “At which point I was planning to just set it on random and just tick through questions as they came which would hopefully pull up any drastic holes that I can then try and fill”*

### Learning

Various factors affected the learning stage, mainly the resource used, the training rotation and the nature of assessment.

### Resources

Reading was described as *“quite laborious”* and most participants preferred to study from *“YouTube and webinars”* describing themselves as “*visual*” learners. P3 and P7 felt reading textbooks to be *“pointless”* and *“useless”* because it was *“not going to be retained”*. They felt learning is best *“reinforced with practice questions”* and they knew this since medical school or school times.

The criticisms for books were varied depending on the participant’s preferences, most did not prefer reading books stating that clinical experience or self-made notes were more superior learning resources. For example, P3 states that “*you just go to find a different answer in every single book”* when attempting to understand certain clinical concepts that are best learned experientially due to some degree of uncertainty or subjectivity.

Some trainees found books useful for Part 2 of the exam which collated information well for them under a clinical presentation. P3: *‘’Part Two was where I did use a book, which was helpful just to be able to look at the end of the topics that the book was covering, and sort of have that like list of things that I needed to go for it and I mean that was useful in terms of books.*’’ P3 values books for their structured, organised content, using them as guides to navigate the material efficiently. However, their engagement with the book is limited to specific learning needs for the Part B FRCS (e.g. collating information against a clinical presentation), suggesting they use it more as a reference tool than for deep, comprehensive study.

One trainee invested significant time in critiquing various resources before making a choice on the best resource to use. *P2: “I spent a lot of time exploring resources and then sort of choose which I want to go with. I do sort of test them out a bit. So, I have got some textbooks that I’ve maybe read one chapter off, because the way the person writes, I just don’t get with it or might be able to get one particular topic from them”.*

P5 described how ready-made revision notes were similarly *“useless” because,* “*revision notes are only really good if you’ve assimilated them yourself, written them yourself”*. Instead, participants preferred more active learning strategies such as *“spider diagrams”* and making summary notes with multiple resources used to collate information into *“one side of A4 paper”.* This learning strategy was popular with some participants who preferred to collate information from multiple sources into one set of self-made notes for ease of access and revision of the information later*.*

*P2* values efficiency, prefers visual aids, and actively engages with resources to condense complex information for quicker review. *“I have redrawn some of the pictures and condensed it and just made an image with the key facts on one side of A4 in colour. So that I can look back over the approach in five minutes rather than spending half an hour reading through it in a book, so its distilling down a resource”*.

Others were also driven by a desire for efficient, optimised study techniques and taking a self-directed approach to finding tools that support their exam-focused learning goals. P3: *“I did use a flashcard app called Anki. And it's got this [spaced repetition] algorithm, I don't know how I came across it, but I guess I must have been looking at how to revise efficiently pass an exam efficiently”.* The trainee’s primary motivation appears to be passing exams efficiently, indicating that their learning strategy is goal-oriented, with a focus on exam preparation.

### Rotation

Another important factor which affected learning was whether the participant had rotated through the relevant speciality. P1 and P6 were *“really lucky”* as they rotated through all subspecialities by exam time and thus did not need to organise their own experience for the niche areas of paediatrics and spines. The more junior participants struggled with the teaching not coinciding with the speciality they were rotating through; *“I was rotating on knees and the regional teaching was on foot and ankle”*, this had *“a negative effect overall, because I didn’t learn either”.*

P5 took a more passive approach, relying on learning through experience on the job rather than actively seeking out study material after work.

*P5: “I never came home from work being like right I need to read a topic today. But it was all like just acquired from doing the job and then focused during the exam revision.”* In contrast to P5, P1 had the *“philosophy…to study what I am doing on the job, as those are the things that I am seeing in work and the things that I should be reading about*”. P5 viewed learning as something that occurs naturally through experience and later focuses their study efforts for exams. Meanwhile, P1 believes in continuously learning by studying job-related topics as they arise, indicating a more consistent and continuous approach to knowledge building.

### Assessment

Part A and Part B were considered by the participants to be very different assessments that needed distinct types of learning. For Part A, *“rote learning, pairing concepts together, random conditions with the genes that are affected, like all these random concepts that are completely arbitrary”* was the predominant experience*.* For Part B, more active learning strategies were used that included *“condensed notes to make a script for each thing”*, *“the script, like being an actor”* so that they “*could speak it back, because it’s a verbal exam”.* Additional techniques included elaborative explanation, supportive explanatory summary notes, spaced repetition, mind maps and conceptual integration. Participants were familiar with the importance of active learning from their early school years.

Tables 5 and 6 summarises the properties of learning and the factors which affect these properties.

**Table 5:** Properties of learning and influencing conditions

Property	Influencing factor				Example Quotes
	**Learner factors**	**Assessment Factors**	**Resources Factors**	**Programme factors**	
Type	Experience Learning style	Exam format Subject matter	N/A	N/A	*P1: “And I've learned through med school that doing your assessments or your reading always went hand in hand with questions”* *P3: “I did a lot of my revision through YouTube. Just listening to people talk about things then making my own notes. Because you can go over and over again”* *P2: “I spent a lot of time exploring resources and then sort of choose which I want to go with. I do sort of test them out a bit. So, I have got some textbooks that I’ve maybe read one chapter off, because the way the person writes, I just don’t get with it or might be able to get one particular topic from them”*
Depth	Judgement	Mock Exam	N/A	Power balance Rota Service provision Disorganisation of learning programme	*P2: “Whereas the feedback (from the Mock UKITE exam) allows me to see where I haven't gone to depth. So, it allows me to then dig down into the areas which are missing. And which I kind of omitted or might have misperceived. So, it kind of allows me to correct errors in learning”* *P3: “yeah and some of the information as well regarding guidelines or say where would you put a pavlic harness, how much flexion of the hip you would do, when would you follow up the patient, some of that if you read in books you just go to find a different answer in every single book”*
Intensity	Motivation Self-image Priorities	Time left to the exam	N/A	Supervisor expectations Relaxation of service commitment	*P5: “That actually started consciously, (nearer the exam), I need to do revision most nights, so probably four months of working hard and maybe one month before that just doing it occasionally”*
Setting	Trainee’s preference Organisation skills Motivation Personal circumstances	Subject matter Exam format (Part B)	N/A	Organisation of the rotation Service provision Power balance	*P2: “But then to help me contextualise that, I then go to a clinic”* *P1: “So throughout training, I’ve made really in-depth notes”* *P3: “…get from an actual clinical experience rather than a book which is impossible. You can’t get that from a book”* *P2: “It’s another power balance as well, and with different consultants it’s to do with how comfortable they are as well in that position. Where the interaction is viewed as a challenge in that they are being criticised to kind of change or a challenge in terms of expanding both people’s learning.”*
Active/passive	Experience Reflexivity	Subject matter Time left for exam Exam format	Wordiness of resource Technology	Setting (experiential versus home) Disorganised rotation Power balance Service provision	*P2: “So that I can look back over the surgical approach in five minutes rather than spending half an hour reading through it in a book, so its distilling down a resource”* *P5: “I basically wrote flashcards with each thing. Just had conditions on and then just have like some of the key things you need to mention for that condition. There’s no point learning anything new, but you just need to have some way of just getting it to tick over in your mind”* *P3: “I tried to get most answers within one page or topics on one page”* *P5 “never came home from work being like right I need to read a topic today. But it was all like just acquired from doing the job and then focused during the exam revision.”*

**Table 6:** Factors influencing evaluations of outcomes

Property	Influencing factor				Example Quotes
	**Learner factors**	**Assessment Factors**	**Resources Factors**	**Programme factors**	
Conformity	Perceived utility Realism Pragmatism Perceived fairness Emotion Perceived knowledge level Trust/Distrust Personality Growth mindset Fixed mindset Self-image	Legitimacy	N/A	Legitimacy Feedback style Personality Consistency of feedback Specificity of feedback Helpfulness of feedback Growth mindset Fixed mindset	*P1: “But I am not sure how much I self-assess, maybe I do, but I think you need to know the benchmarks. You need to then reflect on where you are and enough people give you feedback that you kind of know where you are, because they told you, not because you know necessarily. So, I don’t feel like there was a lot of space or need for self-assessment”* *P6: “Whereas I think if I was chatting to a supervisor or to a potential supervisor or an earlier supervisor and they had concerns or worries, that's when I would take that in much more. That's much more valid to me than the things on ISCP”* *P6: “So, it's not necessarily a personal fault, but it's a thing that you need to try and be aware of and somehow mitigate but You're not always going to manage that”* *P6: “So, it was a third party that gave it so I felt that I could have a frank conversation with the boss that was talking me through the feedback and that we came to the conclusion that I have to take things on board which is obvious, and that adjusting how you come across is part of the job “* *P6: “how I can make it any better” and “feels like if you redo that, six months or whatever, that you’re going to get the same thing, you haven’t gone forwards anywhere, they’ll still have the same opinion of you. You’ll still not have gotten better. So, it’s just a lose-lose situation”*

### Evaluating outcomes

The Multiple-Choice Question (MCQ) exam was perceived as vague by participants, and thus they did not feel the test evaluated their knowledge or abilities accurately. Participants stressed the importance of logical reasoning skills when solving MCQs and highlighted the need to avoid inhibiting problem-solving skills due to perceived knowledge deficiencies. Participant seven: “*I would assume that there was a gap in my knowledge and that was a fatal mistake”.*

Participants noted that some MCQs had remarkably similar answer choices. They criticised some MCQs as vague with various options being plausible where they *“could quite happily do three of these because they are all right”.* Similarly, P6 stated *“I don't think they're vastly valid assessments”.* P7 passed the exam eventually but “actually *did less revision”* on their last attempt. They believed they *“passed it through technique”.* This suggests that part of SDL involves understanding the assessment process particularly how MCQs are phrased and what they aim to test.

In contrast, the VIVA exam, which integrated all facts under a medical presentation, was viewed favourably by participants allowing for more authentic assessment of their knowledge and understanding.

Self-assessment was considered not applicable to training in Orthopaedics by some participants. For example, Participant one was “*not sure how much to self-assess… to assess your own learning…. So, you really need people who you can bounce things off, and to say to them I am achieving the right level?”.* P1 states that self-assessment is likely to be less accurate than external assessment by a senior colleague who has more experience, as they have undergone similar training and exams and thus have valuable insights to offer. P1: *“ I wouldn't wholly go against what they said”.* Similarly, P6 stated *“I think if I was chatting to a supervisor … and they had concerns or worries, that's when I would take that in much more”.* This indicates that some trainees see the learning process as one that involves guidance from experienced mentors.

However, occasionally, feedback was not accepted if participants felt it was unreliable with no clear method of improvement offered. P6: *“how* I *can make it any better”* and it *“feels like if you redo that, six months or whatever, that you’re going to get the same thing, you haven’t gone forwards anywhere, they’ll still have the same opinion of you. You’ll still not have gotten better. So, it’s just a lose-lose situation”.* P6’s statements reflect a strong desire for self-improvement, coupled with frustration and concern about stagnation and the negative perceptions of others regarding their growth.

### Self-advocacy and negotiation

The complexities of interpersonal relationships and the power balance between supervisors and their trainees were apparent during clinical sessions. Trainees were often faced with the challenge of needing to advocate for oneself for a learning opportunity while at the same time not seeming too assertive or “pushy”. At one stage P7 *“had to fight for”* allocation to a spinal surgery rotation as *“if you never worked for a spinal firm, it can be very difficult [to answer questions on spines in the exam].”* Despite the requirement for this, he did *“feel unpopular and silly”.*

Some trainees described that certain requests or actions, such as asking to operate on a case or questioning their supervisors about specific topics, may not be well-received if there are high service pressures. *P2: “ I felt asking those things (e.g. to operate on a case) and exploring those topics (might not be) emotionally well received, (especially) when service requirements do not allow that because there’s a high patient turnover. And because I'm quite sensitive to all those aspects, I will tend often not to push and say, I need to do this (operation)”.* Service pressures meant that P2 needed to be mindful of the situational appropriateness of asking for a learning opportunity.

On the other hand, it was important to be proactive and still put oneself "out there," P1: *“if you don’t put yourself out there…people forget about you or walk over you deliberately or unintentionally, and you will find you’re not the person they call when they have an interesting thing, they need you to do, or they’ll forget about you*.” This highlights a concern that without active participation, individuals may become invisible or overlooked by their peers or colleagues. There is also the concern due to perceived risks associated with self-advocacy, learners could fall into a “*medical student role of not having active involvement*” leading ultimately to disengagement.

Participants felt uncomfortable when discussing assertiveness as a concept. *P1: “You don’t want to be aggressive; you want to be engaging”*. They perceived it to potentially lead to negative outcomes as they could be perceived as pushy. One female participant commented how gender can influence assertiveness depending on the cultural expectations of a society. *“So, men are kind of allowed to be a bit more aggressive in the general population that is that is kind of viewed as normal. And women who is assertive is often referred to as bossy.”*

Participant 1 valued interactions with individuals *“that make you feel more like a peer rather than a student”*. Thus, fostering a sense of mutual respect, allowing P1 to feel more confident in contributing to discussions and asking questions. The “power balance” was implicated as well, and whether *“the interaction was viewed as a challenge [by consultants], in that they are being criticized to kind of change [management] or a challenge in terms of expanding both people’s learning”.* The power dynamics between consultants and trainees was seen as an influential factor in how feedback or interactions were perceived by both parties.

Self-advocacy and negotiation also played a key role during the discussion of feedback, overlapping with the previous section where end of placement feedback was discussed between trainer and trainee. Participants explored how they could align their perspectives on negative feedback and find common ground with the assessor, rather than entirely oppose the evaluation. P6: “*so I felt that I could have a frank conversation with the boss that was talking me through the feedback and that we came to the conclusion that I have to take things on board which is obvious, and that adjusting how you come across is part of the job”.* This highlights the importance of acknowledging feedback and showing a commitment to acting on it, demonstrating a continuous process of self-improvement.

Table 7 summarises the factors which influence how participants negotiate training opportunities and feedback with their training with supervisors and programme directors.

**Table 7:** Factors influencing assertiveness when negotiating learning and feedback

**Property**	**Influencing factor**		
	**Learner factors**	**Programme factors**	Example Quotes
Vigor (Assertiveness)	Expectations Assertiveness Self-perception Beliefs Perceived barriers Reputation Priorities Learning contract	Resources Rotations organization Trainer resources Trainer ability Expectations Perceptions Priorities Learning contract	P*: “So, men are kind of allowed to be a bit more aggressive in the general population that is that is kind of viewed as normal. And women who is assertive is often referred to as bossy, do it because those stereotypes are held up to what people can do in terms of the culture. Now, equally there's slightly different risk areas with that because again, a lot of the theatre staff are female.” *P6: “Adjusting how you come across and how you discuss cases is part of the job. It’s part of the game”* *P2: “But there’s a bit where asking those things and exploring those topics [might not be] emotionally well received, [especially] when service requirements do not allow that because there’s a high patient turnover”* *P1: “I think if you don’t have a sort of the right kind of exposure, then it becomes really hard to self-direct learning”* *P6: “You’re not actually telling me how I can make it any better. So, you almost feel like if you redo that, six months or whatever, that you’re going to get the same thing, you haven’t gone forwards anywhere. And no one wins in that situation. They’ll still have the same opinion of you. You’ll still not have gotten the better. So, it’s just a lose lose”* * P2: "The whole point is that I am doing this so that I am proficient. If I have not reached that point, I want to be extended. And by having that discussion, it then freed up opportunities for me to actually do procedures so that I can reach a level of competence"* P5: “I remember when I didn’t do particularly well on the exam. Prior to the one that I sat and ***** sent me a message saying I didn’t do as well as we thought, we might not be able to put you up for the exam for next year as well. I didn’t try to do it because I was trying to knock out a 100 Hip Replacements in a year, so I don’t have to do any more Hip Replacements. Again, that wasn’t my priority at the time” *P1: ‘’So, I can’t see how I can separate my opinion on where I’m at, from other people’s opinions where I’m at, because my opinion where I’m at is informed by what they say to me. And I wouldn’t wholly go against what they said’’* *P2 “And because I'm quite sensitive to all those aspects, I will tend often not to push and say, I need to do this”* *P1 “particularly people that make you feel more like a peer rather than a student, you can bring stuff with them, making you feel valued, making it sound like your question is a valid question”* * P2: "But there's a mixture needed as well because it's very easy to fall into a sort of medical student role, of not having active involvement"* * P2: ''It's another power balance as well, and with different consultants it's to do with how comfortable they are as well in that position. Where the interaction is viewed as a challenge in that they are being criticised to kind of change or a challenge in terms of expanding both people's learning"*

## Discussion

The primary assumption underlying the research question was that no programme would be capable of providing exhaustive training and guidance. Consequently, learners would need to take on a self-directed approach to acquire certain essential knowledge. The findings showed that self-directed learning in a training environment is impacted by the training programme, yet learners kept a degree of autonomy.

### Model of SDL in Orthopaedic trainees

**Fig. 3:**
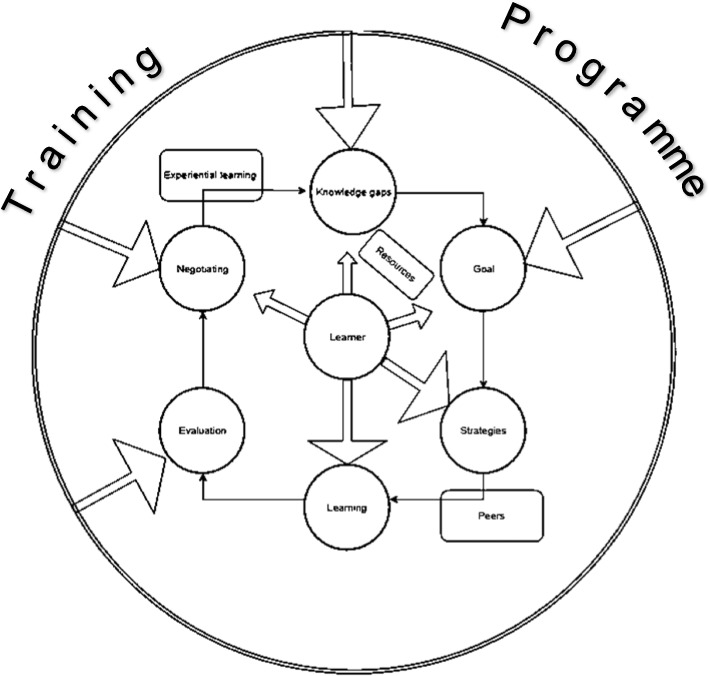
Model For SDL in Orthopaedics Trainees

Constructing the model involved careful consideration of the training context. Figure 3 illustrates participants' experiences of the significant influences from their training environment. Goal setting was initially challenging due to the high knowledge standard required, making external guidance necessary. Experiential learning models, like Cutrer et al.'s [11], were relevant initially but lacked consideration of the instructional context. Knowles's SDL model emerged as more applicable, accounting for the training context.

The model in Fig. 3 portrays participants' perceived lack of control at certain SDL stages (goal setting, evaluation, negotiation, identifying knowledge gaps), alongside increased autonomy in others (selecting strategies and learning). It resembles Tompkins and McGraw's [37] negotiated learning contract, with negotiation occurring after evaluation due to infrequent collaboration between Orthopaedic trainees and supervisors. Tompkins and McGraw stressed the importance of supervisors allowing autonomy, while clarity and transparency in the learning contract's first stages prevent mismatched expectations.

Participants, like participant two and five, negotiated with programme directors due to differing priorities or doubts about achieving training requirements. Assessment factors and available resources, including those external to the programme, significantly influenced SDL. These factors are further discussed in the next sections.

### Resources

Participants stressed the importance of avoiding certain resources such as poorly written books or those containing scientific errors and outdated information. Digital resources like YouTube videos, podcasts, video lectures, and webinars were commonly preferred. Professionally written textbooks with clear illustrations and detailed descriptions were also favoured. All participants subscribed to the Orthobullets.com online revision programme, which provided reading material and multiple-choice questions (MCQs) covering the entire curriculum over a few months, incorporating spaced repetition algorithms.

The vast number of learning aims published by the ISCP posed a challenge for learners to navigate [4]. Hence, learners primarily relied on an American resource, Orthobullets.com, due to its convenient overview of the curriculum. King [21] highlighted the challenge of time management in learning and the need for learners to design a comprehensive plan. Orthobullets.com served as a "Master plan," offering a structured curriculum overview with milestones over a 21-week period. Its embedded spaced repetition algorithms aided long-term retention and addressed time management challenges.

Teaching on topics unrelated to current rotations had adverse effects on some participants. Asynchronous learning technology could enhance SDL by allowing participants to access learning materials at their convenience. Self-paced learning has been linked to improved memory retention [30], and online software with spaced repetition algorithms can further optimize memory retention [19]. Interactive online platforms with tutor support, such as asynchronous blog discussions, can facilitate learning.

### Selecting strategies

Participants continually reflected on their study strategies, adjusted them when necessary, and maintained a growth mindset. Participants acknowledged that they would never "know enough" and viewed themselves as being on a continuous learning journey in a growth mindset that kept them motivated [36]. Feedback from mock exams was seen as an opportunity to identify and correct learning errors rather than a failure.

Participants employed various strategies to maximize retention, such as supportive notes, practice testing, spaced repetition, and elaborative explaining [20, 35]. Peers played a significant role in helping participants select effective study strategies.

The effectiveness and widespread use of active recall and spaced repetition with Anki flashcards is well-documented, providing a valuable approach for teaching complex topics [29]. Dunlosky et al. [15] emphasised that using one-sided A4 paper for note-taking enhances organisation and facilitates learning by promoting a linear structure that improves clarity and flow. This format allows students to adopt consistent layouts, such as bullet points and hierarchies, making it easier to locate specific information during revision. The restriction to one side reduces clutter, encouraging selective summarisation, while white space and visual segmentation helps students navigate their notes. Additionally, one-sided notes simplify the review process, support self-testing, and improve portability, ultimately leading to a more effective study experience and better retention of information.

### Evaluating outcomes

Participants critically assessed various facets of assessments, showcasing forethought and independence in their learning decisions. Interventions aimed at helping learners understand the assessment process and its limitations were considered more effective than targeting "elevated risk" learners who are presumed to lack knowledge.

For example, participant seven stated they eventually passed the MCQ through adjustments to their test taking and problem-solving technique and not knowledge. Through a think aloud exercise with a colleague, the participant appreciated the process required to arrive to the correct answer. Multiple studies show that conscious thought enhances problem solving. In the research, participants who verbalised their thought process showed better performance [2]. The inclusion of “higher order” MCQs makes problem solving skills an essential component to success in the exam. Therefore, learners should be encouraged to use their logical reasoning skills and not inhibit them due to perceived knowledge deficiency.

### Self-advocacy

A key aspect of self-direction is the ability to seek out opportunities and advocate for oneself, while maintaining a tactful approach. This concept can be termed "strategic self-advocacy”. Several factors influenced strategic self-advocacy, including case complexity, power dynamics, interpersonal relations, and competing service provisions. Interpersonal relations and rapport were believed by participants to be crucial for successful negotiation. The Rapport Management (RM) framework, considering face sensitivities, social rights and obligations, and interactional goals, can empower learners to negotiate effectively [28].

Feedback delivery could benefit from the RM model, ensuring non-judgmental, non-threatening feedback in a psychologically safe environment. Learner-centred feedback methods empower learners to self-assess, learn from mistakes, and take greater responsibility for their learning. Mutual respect helps trainees feel validated, appreciated, and more engaged in the learning process. This suggests that creating an environment where learners feel that their input is respected and their questions are legitimate enhances their sense of value and encourages active participation.

Additionally, the power dynamics between consultants and trainees emerged as a key factor in how feedback was experienced by trainees. For feedback to be truly effective, consultants should aim to provide more constructive, balanced feedback that encourages openness and development rather than create defensiveness. Achieving this balance is crucial to fostering a culture of continuous learning, where feedback serves as a platform for professional growth rather than initiate trainee disengagement from feedback that is perceived to be unduly negative or unfair.

### Strengths and limitations

This study elicited insights from seven Orthopaedic trainees about SDL. The focus on trainee perspectives may introduce bias, particularly in recommendations focusing mainly on challenges faced by trainees. Participants acknowledged difficulties in accurately evaluating their progress, particularly in the early years of training. Over-analysis of participant statements could lead to inaccurate conclusions on effective learning methods. Additionally, learners’ feelings of self-efficacy with a particular style or method of learning or instruction do not necessarily equate to actual efficacy. Incorporating input from consultant orthopaedic surgeons would provide a different perspective and in turn a more holistic understanding of SDL in an Orthopaedic training context.

## Conclusions

Although the United kingdom’s Trauma and Orthopaedic curriculum is described as trainee led and SDL is encouraged, in practice Orthopaedic trainees felt limited autonomy with the various stages of SDL due to external factors including their level of experience and the training environment. Trainees’ experiences around self-advocacy highlighted the importance of a collaborative and supportive learning culture emphasising dialogue, receiving high quality and fair feedback, demonstrating willingness to act on it, and openness for successful progression. Learners should be given reassurance that their self-advocacy or proactivity would not be interpreted adversely by their trainers. This is to avoid dissuading learners from seeking learning opportunities and inadvertently falling into passive roles due to the risks they perceive to be associated with self-advocacy. Further studies should incorporate the perspectives of consultants to provide a more holistic understanding of the subject.

## Data Availability

Interview transcript data can be made available by contacting the primary author on a.abdelmaksoud@nhs.net.

## Abbreviations

FRCS: Fellowship of the Royal Colleges of Surgeons
GMC: General Medical Council
ISCP: Intercollegiate Surgical Curriculum Programme
JCST: Joint Committee on Surgical Training
JCIE: Joint Committee on Intercollegiate Examinations
MaxQDA: Max Weber Qualitative Data Analysis
MCQ: Multiple Choice Question
PBL: Problem Based Learning
RM: Rapport Management
SAC: Surgery Advisory Committee
SBA: Single Best Answer
SDL: Self Directed Learning
SMART: Specific, Measurable, Achievable, Realistic, Timely
SRL: Self-Regulated Learning
ST3: Speciality Training Year 3
UK: United Kingdom
UKITE: UK and Ireland In-Training Exam
VIVA: Viva Voce, Latin for by living voice
